# *ZNF804A* Genotype Modulates Neural Activity during Working Memory for Faces

**DOI:** 10.1159/000344001

**Published:** 2013-01-04

**Authors:** David E.J. Linden, Thomas M. Lancaster, Claudia Wolf, Alison Baird, Margaret C. Jackson, Stephen J. Johnston, Rossen Donev, Johannes Thome

**Affiliations:** ^a^MRC Centre for Neuropsychiatric Genetics and Genomics, Cardiff University, Cardiff, Bangor, UK; ^b^Neuroscience and Mental Health Research Institute, Cardiff University, Cardiff, Bangor, UK; ^c^School of Medical Sciences Neuroscience, School of Psychology, Bangor University, Bangor, UK; ^d^Wolfson Centre for Cognitive and Clinical Neuroscience, School of Psychology, Bangor University, Bangor, UK; ^e^Laboratory of Molecular Psychiatry and Pharmacology, Institute of Life Science, School of Medicine, Swansea, UK; ^f^Department of Psychology, Swansea University, Swansea, UK; ^g^Department of Psychiatry and Psychotherapy, University of Rostock, Rostock, Germany

**Keywords:** Emotion, Dorsolateral prefrontal cortex, Genetic imaging, Schizophrenia, Working memory, *ZNF804A*

## Abstract

**Background:**

Genetic susceptibility to schizophrenia (SZ) has been suggested to influence the cortical systems supporting working memory (WM) and face processing. Genetic imaging studies link the SZ risk variant rs1344706 on the *ZNF804A* gene to psychosis via alterations in functional brain connectivity during WM, but no work has looked at the effects of *ZNF804A* on WM with face-processing components.

**Methods:**

We therefore investigated healthy controls that were genotyped for rs1344706 with a face WM task during functional magnetic resonance imaging. We suggested that variation at the rs1344706 locus would be associated with similar alterations as patients previously tested using the same WM task for faces.

**Results:**

The rs1344706 risk allele was indeed associated with altered activation in the right dorsolateral prefrontal (rDLPFC) cortex. We established that the rDLPFC was activated in a task-dependent manner, suggesting that the differences in activation between rs1344706 genotype groups reflected alterations in task processing. Furthermore, we demonstrated that the rDLPFC region showed significant volumetric overlap with the rDLPFC which had previously been reported to be altered during task processing for patients with SZ.

**Conclusions:**

The findings support an association between rs1344706 and alterations in DLPFC activity during WM for faces. We further suggest that WM for faces may be a useful intermediate phenotype in the investigation of genetic susceptibility to psychosis.

## Introduction

Genome-wide association studies have identified a locus on the *ZNF804A* (rs1344706) as a well-supported risk variant for schizophrenia (SZ) and a broader spectrum of clinical phenotypes [1,2,3,4,5]. In order to quantify the potential functional effects of variants of genome-wide association studies such as *ZNF804A*, functional magnetic resonance imaging (fMRI) is used to study how genetic architecture contributes to neural systems. This method may help to establish how risk variants may modify the neurobiological pathways that are disrupted in psychiatric populations [6,7].

Working memory (WM) and facial processing are recognised as heritable deficits in SZ [8,9]. WM and face processing are also implicated as the biological basis for neuropsychiatric symptomatology [10,11,12,13]. Patients with SZ show alterations in task processing for WM and emotional faces as revealed by functional neuroimaging [14,15]. Relatives of SZ patients (familial high-risk groups) also display similar alterations [16,17,18]. The rs1344706 genotype (T = risk allele) is associated with alterations in functional connectivity between prefrontal and inter-hemispheric prefrontal/hippocampal networks in healthy controls during WM [19,20,21], face processing and resting state [22]. The functional effects of the rs134407 variant may extend to a broad range of cognitive phenotypes such as social cognition [23,24] and attentional networks [25,26]. ZNF804A may influence cell adhesion [27] and regulate expression of other genes [28], whereas the rs1344706 variant may have a functional role in the transcription of the *ZNF804A* gene [29]. However, it is not understood how the rs1344706 variant influences complex neurocognitive phenotypes, with emerging evidence suggesting the variant has little/no effect on macroscopic cortical structure [30,31,32].

The rs1344706 variant may modulate prefrontal cortical functional connectivity implicated in the WM process [19,20]. However, the robust alterations in prefrontal neural activation during WM observed in schizophrenic patients [33,34,35] and first-degree relatives [16,17,18,36] were not associated with the rs1344706 allele in SZ patients, first-degree relatives or healthy controls [21]. That said, both heritability (twin studies) and SZ-related genetic risk score (cumulative total of SZ risk alleles) are both significant predictors of neural activity in the dorsolateral prefrontal cortex (DLPFC) during WM [37,38].

In order to further explore the effects of the rs1344706 genotype on the WM network, we added a face-processing component to a WM task. We suggest that the addition of face processing to WM items will recruit a specific neural network and a novel context to probe for functional effects of the rs1344706 variant. Studies using affective WM stimuli reveal that SZ patients have relatively intact limbic function (amygdala activity) in response to emotionally valenced items, but show altered activity in the DLPFC [39] that may reflect a deficit in emotion recognition [40]. During WM for faces, SZ patients failed to utilise conventional neural resources (hypoactivation in the right PFC) and, instead, recruited a contralateral homologue (hyperactivation in the left PFC and sensory cortical regions) to manage the WM demands [41].

In the present study, we test the hypothesis that the rs1344706 risk variant on the *ZNF804A* gene will modulate brain activation during face WM in healthy controls. We use the same WM task using faces (previously described [41,42,43]) to probe for the neural effects of rs1344706 on WM for faces in healthy individuals. We suggest that the specific cortical architecture involved in face WM [41,42,44] may provide increased sensitivity and specificity. More specifically, we predict that the rs1344706 variant will modulate WM processing for faces in a manner that reflects the alterations first observed in schizophrenic patients [39,41].

## Materials and Methods

### Participants

Forty-three healthy subjects of European Caucasian descent with no family history of neurological or psychiatric illness where recruited for the study. Participants provided written consent prior to the study, which was approved by the School's Ethics Committee. Participants from each rs1344706 genotype group did not differ in education, age, sex and handedness or WM capacity (table 1), all of which had normal or corrected vision. Data were from a subsample of participants from a larger genetic imaging study [45], for which *ZNF804A* rs1344706 genotype data were available.

### ZNF804A Genotyping

Subjects were genotyped for the *ZNF804A* rs1344706 G/T SNP. Genomic DNA was extracted from venous EDTA samples [Invisorb® Blood Giga (Invitek GmbH, Germany)]. Amplification of the target sequence on the *ZNF804A* gene was carried out using PCR (*ZNF804A* forward: 5′-CCACTAGCAACAACTCCCTCA-3′,*ZNF804A* reverse: 5′-TCTAGAGTCATGCAGGCACA-3′). The following PCR protocol was used: 10 min at 95°C, followed by 35 cycles of 94°C for 30 s, 60°C for 30 s and 72°C for 30 s, and by 72°C for 2 min. The amplicon was visualised on a 2% agarose gel stained with SYBRsafe (Invitrogen, UK) under UV light, following separation at 100 V in Tris-borate electrophoresis buffer. The PCR product was digested with the BfuCI restriction endonuclease (New England Biolabs, UK) and reaction buffers at 37°C for 16 h. The resulting digested samples (TT genotype = 216- and 186-bp fragments, GG genotype = 186-, 154- and 62-bp fragments) were separated on a 2% agarose gel as previously described and scored for genotypes (GG = 11, GT = 21 and TT = 11). Hardy-Weinberg equilibrium was checked with χ^2^ = 2.63, p > 0.1.

### Stimuli

Six adult, male greyscale Ekman face images each displaying happy, neutral or angry expressions were used. Each image covered approximately 1.43° × 1.36°. Scrambled greyscale face images selected at random were displayed to cover the face locations when participants encoded less than 4 faces. All stimuli used were evaluated for appropriate emotional valence/expression [43].

### WM for Face Paradigm

**Fig. 1:**
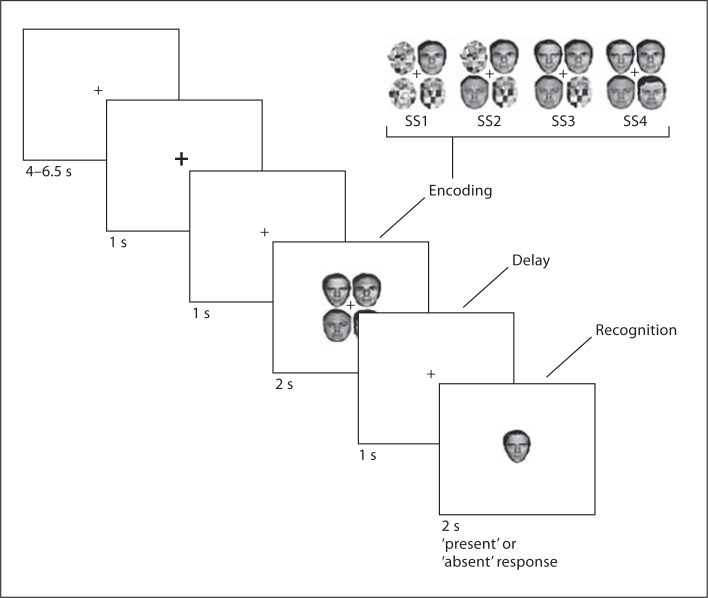
The emotional face WM paradigm. Dynamic of the trial and session structure. After a jittered fixation interval, participants were given 2 s to encoding emotional faces (1-4 faces, empty array components were replaced with scrambled faces). Participants then experienced a 1-second delay followed by a 2-second interval in which to respond.

In an event-related design, we investigated visual WM for faces and task-related brain activity through manipulation of facial expression (happy, neutral and angry) and number of faces to be remembered (load 1, 2, 3 and 4). Faces were presented at randomly alternating locations in a 2 × 2 array in the center of the screen, and the center of each image within the matrix was positioned at a visual angle of approximately 1.271^o^ from fixation to ensure that the face display was in direct line of sight (fig. 1). Each of the 12 conditions consisted of 16 trials divided into 8 match and 8 non-match trials. Participants indicated whether the single probe face presented after the array was ‘absent’ or ‘present’. Facial expression and number of faces varied randomly between trials and face expression was kept constant for each individual trial. All trials started with a fixation (2,000 ms) towards a central cross that served as a baseline predictor. This was followed by a 2-second presentation of the face array, a 1-second delay and the probe face, where participants had to indicate either a match or non-match response. There were 192 trials distributed over 4 runs of 48 trials to minimise fatigue effects. Trials lasted less than 14 s (343 volumes, 2 s TR, WM sessions were 686 s, covering all 48 trials). The task was generated and responses were recorded using E-Prime software (version 1.1; Psychology Software Tools, Inc., USA). WM capacity for faces was measured by individual Cowan's K values for each emotion and load condition [Cowan's K values = array size × (hits – FA)], where FA = false alarms [46].

### Imaging Procedure

We acquired fMRI data (T2*-weighted echo planar imaging sequence; TR = 2,000 ms; TE = 40 ms; matrix size = 96 × 96; FOV = 256 × 256 mm; voxel size = 3 × 3 × 3 mm; 90° flip angle; 20 axial slices; 5 mm slice thickness) on a 1.5-tesla Philips whole-body MR scanner. Imaging data analysis was performed using the BrainVoyager 1.9.10 software (Braininnovation, The Netherlands). Functional images were co-registered with the structural 3D image, spatially normalised to the Talairach system [47] and resampled at a voxel size of 1 × 1 × 1 mm. Functional images were scan time corrected using sinc interpolation, 3D motion corrected using trilinear interpolation, spatially smoothed (8-mm gaussian kernel) and filtered into the time domain using high-pass filter (3 cycles per time course; 0.0044 Hz). Each WM session acquired 343 volumes, the first two of which were discarded to reduce potential T1 saturation effects. The 43 participants each completed 4 WM sessions. The resulting 172 single-subject design matrix files were incorporated into a general linear model (GLM) analysis with 20 predictors, including fixation (1), conditions for all correct trials (12), all error trials (1) and predictors derived from the head motion correction for each subject (6). All but the motion predictors were convolved with a two-γ haemodynamic reference function. The predictors from all 4 sessions were concatenated into a single predictor per subject. At the first level, we estimated β values for the remaining 14 predictors [12 conditions: 3 emotions (happy, neutral and angry) × 4 WM loads] and separate predictors for modelling baseline activity (1) and all error trials (1) for each participant with the least-square estimate of the GLM. The estimated β values were entered into a random-effect GLM to test for potential effects of the rs1344706 genotype.

### Analysis of Neuroimaging Main Effects (Emotion, Load and rs1344706)

*ZNF804A* rs1344706 effects were tested with a 3 × 4 × 3 random-effect ANCOVA with the factors emotion (happy, neutral and angry) and load (1, 2, 3, 4) as within-subject factors and rs1344706 (GG, GT and TT) as between-subject factor. Main effects and interactions were computed separately for each factor. Cluster thresholds for all analysis (emotion valence, WM load and rs1344706 genotype) were calculated with BrainVoyager QX cluster-level statistical threshold estimated based on a Monte Carlo simulation with 1,000 iterations [whole brain corrected p < 0.05 (p < 0.0001, 4 voxels)]. This threshold technique utilised a level of stringency similar to family-wise error that is needed to control for false-positive results in imaging genetics [48]. In a whole-brain analysis, β values were extracted within clusters that showed significant main effects of emotion for faces, WM load, rs1344706 genotype and potential interactions. Individual β values were extracted as averages for each of the 12 task conditions for all significant voxels.

## Results

### Main Effects of Emotion, Load and rs134476 Genotype (Behaviour)

A repeated-measure ANOVA showed a main effect of face valence on WM capacity (F_(2,84)_ = 5.187, p = 0.008), where capacity for emotional faces was higher than for neutral faces (t_(42)_ = 4.527, p < 0.001). There was also a significant main effect of load on WM capacity (F_(3,120)_ = 40.38, p < 0.001). There were no main effects of the rs1344706 genotype on WM capacity for faces (table 1) and no significant interactions (genotype × emotion and genotype × load; p > 0.5 in both cases).

### Main Effects of Emotion, Load and rs134476 Genotype (Neuroimaging)

Main effects of emotion are documented (table 2). Post hoc tests show these regions are driven by increased activity for emotional faces (p < 0.001 in all cases). Main effect of WM load implicates regions where activation is higher for multiple faces compared to singles faces (table 3). Post hoc analysis revealed a linear increase in neural activity in these regions, as WM load (p < 0.001 in all cases). All regions showing a main effect of load and/or emotion are in line with those previously reported on a subset of the present data [42].

**Fig. 2:**
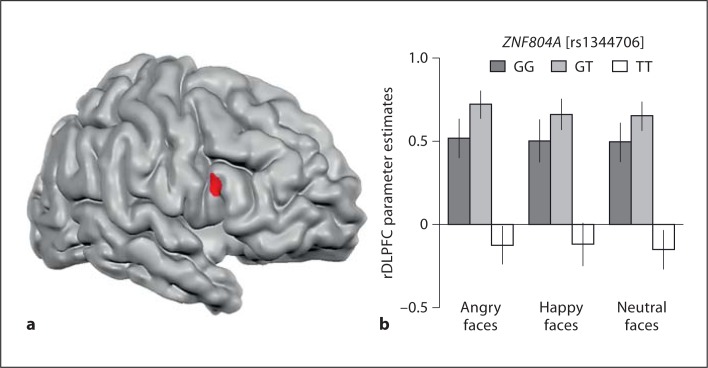
**a** ZNF804A rs1344706 genotype effects on the rostral portion of the right inferior frontal gyrus (rDLPFC) during WM for faces in 43 healthy participants. **b** Parameter estimates for mean neural activity averaged across all 12 conditions in the tasks and separated into rs1344706 genotype groups in 43 healthy controls (TT = risk allele).

There were no significant interactions between *ZNF804A* genotype and WM load or emotion. However, there was a significant main effect of *ZNF804A* genotype on neural activation in the rostral region of the right inferior frontal gyrus (rDLPFC; fig. 2a). Post hoc analysis revealed significant rs1334706 allele differences in rDLPFC during the WM task for faces (GG vs. TT and GT vs. TT; p < 0.01, corrected) but no differences between (GG vs. GT; p > 0.5; fig. 2b; table 4).

**Fig. 3:**
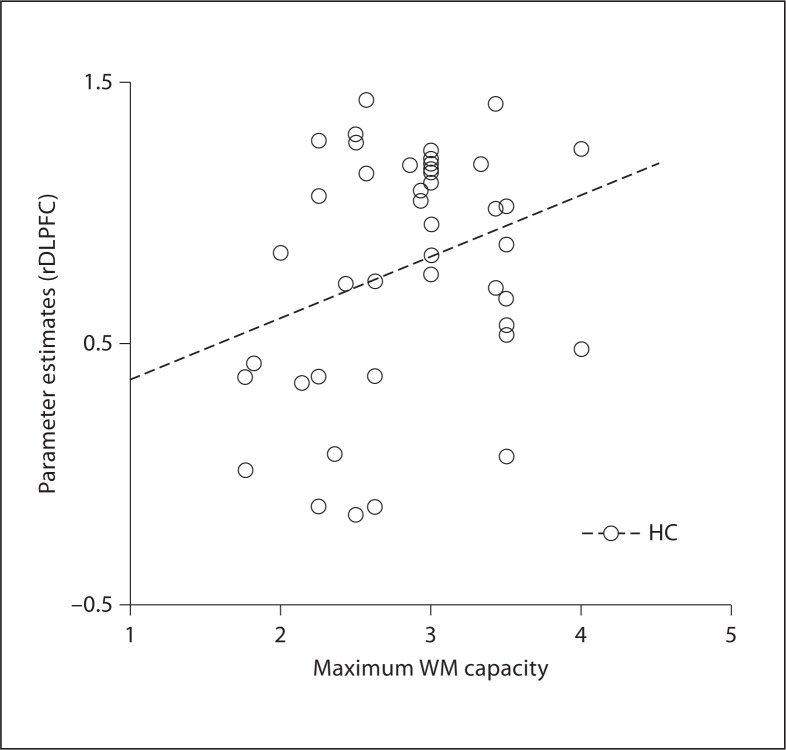
Relationship between rDLPFC parameter estimates (β means across task) and maximum WM capacity (K_max_) for emotional faces in 43 healthy controls (HC).

In post hoc analysis, we discovered that maximum capacity for WM (K_max_) [46] significantly correlated with the parameter estimates of the rDLPFC voxel cluster (r = 0.32, p = 0.037), suggesting the cortical region was recruited in order to deal with task-relevant information (fig. 3).

At this point, it is noteworthy that this region was also significantly under-activated in patients diagnosed with SZ during the same WM task for faces [41]. To help validate alterations in the rDLPFC during WM for faces as a potential intermediate phenotype for SZ, we conducted an exploratory investigation using the rDLPFC cluster (table 2) that was modulated by rs1344706 (fig. 2) in an ROI (region of interest) analysis. Using the time series from a random-effect GLM on 16 individuals (8 healthy controls and 8 cognitively spared patients with SZ), participants met specific inclusion criteria and completed identical methodological protocols. The SZ sample did not significantly differ from the healthy control sample in age, ethnicity, handedness, education and face WM performance [41]. We extracted the β means for all 12 conditions in the rDLPFC ROI and found a significant main effect of SZ diagnosis on activation in the cluster (post hoc). In this analysis, SZ patients showed a reduced activation compared to healthy controls: F_(1,15)_ = 18.06, p < 0.0005. Please note that the ROI time series extracted was from a previous study [41] and is purely illustrative in this investigation. It serves to demonstrate the potential that altered activation in the rDLPFC may be an intermediate phenotype for SZ during WM for faces.

## Discussion

The critical finding of the present study was the main effect of the rs1344706 variant on the ZNF804A gene in the rDLPFC. Neuroimaging methods have identified abnormalities in this cortical region during WM in patients with SZ [49], high-genetic-risk individuals [17,36] and healthy rs1344706 risk allele carriers [19,20,21,22]. These studies have not found an effect of the *ZNF804A* genotype on neural activation during WM. However, we suggest that the addition of face processing and/or higher WM loads may reveal significant deficits in WM in SZ patients and healthy carriers of SZ-associated loci [41,45]. This is the first study to identify alterations in neural activity in *ZNF804A* risk allele carriers during WM. This novel discovery may be due to the introduction of face processing during WM and/or additional WM demand. The inclusion of complex stimuli such as faces may recruit a wider and more complex network of neural resources during WM [39,44,50]. Specifically, the rDLPFC has been implicated in the regulation/attenuation of emotional responses and a neural basis for modulating emotional experience through interpreting and labelling of emotional face expressions [51]. We suggest that the inclusion of the faces in the WM task is responsible for attenuating the effects that *ZNF804A* has on this cortical region. This correlation between maximum WM capacity and neural activity in the rDLPFC supports the notion that differences between rs1344706 allele groups may be attributable to the face-processing component of the WM task. However, we cannot rule out the possibility that the addition of social content to WM stimuli drove the rs1344706 genotype effects. WM for faces may be a potential neurobiological mechanism through which the risk genotype affects a key cognitive function and ultimately may contribute to psychopathology.

It is a subject of ongoing debate what neural processes cause the variability between SZ patients and controls in prefrontal activation during WM [14]. It has been suggested that increased activation can represent neural inefficiencies and the compensatory recruitment of extracortical resources to deal with WM tasks in SZ patients [49]. Patients with SZ may fail to recruit the DLPFC during the WM tasks [52], which may reflect poor integration of neural networks or individual differences in performance and/or motivation [53]. Many confounding factors, such as medication and disease chronicity/duration [52,54,55], may also influence neural alterations in WM processing in SZ patients; therefore, it is important to consider that genetic variability in healthy individuals may not always reflect the same pathological process as in clinical cases [54].

Nevertheless, alterations in the DLPFC have remained a constant observation in neuroimaging studies aiming to quantify the neural correlates of reduced WM capacity in SZ patients. The rDLPFC is also a frequently implicated cortical structure in the putative effects of the *ZNF804A* variant in healthy controls [19,20,21,22]. The face WM paradigm we have previously used reliably recruits the rDLPFC as a component of WM-related architecture [42,44]. It is suggested that DLPFC is implicated in emotional WM by modulating the emotional salience of WM content in order to guide behavioural performance [39]. The previous patient study showed reduced activation in the rDLPFC as key component of aberrant neural activation in SZ patients [41]. We, therefore, presented the effects of the *ZNF804A* risk allele in comparison with the patient data in order to demonstrate that, at least for the rDLPFC activation to this paradigm, the effect of SZ risk is uniform in the direction of hypoactivation. It is certainly encouraging that the prefrontal hypoactivation in the rs1344706-associated cluster was also hypoactive for SZ patients. The results may also help to elucidate clinical impairments associated with rDLPFC dysfunction, such as negative symptoms [13] and social anhedonia [10,11,12,13,39]. Direct comparison of patient data is important in order to determine whether effects observed in individuals at genetic risk for a disorder reflect this risk, or rather the resilience of the unaffected individuals.

Our results conform to neurobiological models of functional abnormalities in SZ patients and high-risk groups, which have widely documented changes in the DLPFC. Our data provide preliminary evidence that the *ZNF804A* risk carriers may fail to maintain a prefrontal network during the WM task in a similar manner to SZ patients. It could be argued that this novel finding was due to the encoding, maintenance and/or retrieval of faces, which will have to be unravelled further in future studies.

Although the mechanisms that mediate rs1344706 effects on WM networks are unknown, sensitive techniques such as functional imaging can thus allow us to trace subclinical effects potentially mediated by variants discovered by genome-wide association studies. The effects of genetic variation are more readily observed in neuroimaging phenotypes compared to behaviour [56,57]. Although the sample size of the present study is within an estimated range needed to observe genetic effects on memory [58], a larger sample may have made the approach more sensitive to additional measures such as *ZNF804A* genotype × load or emotion interactions. However, it is of importance to consider that stringent multiple comparison correction measures were used, suggesting robust findings for the identified region.

Our study adds to the increasing body of evidence for altered rDLPFC function in carriers of the *ZNF804A* psychosis risk variant. Linking altered brain activation with behavioural and ultimately clinical measures is still a challenge, but will be an important enterprise in order to identify the mechanisms that lead from the gene to the disease and fulfil the main hope of psychiatric genetics that it will elucidate new target pathways for clinical interventions.

## Disclosure Statement

None to declare.

## Figures and Tables

**Table 1:** Distribution of demographic characteristics

	rs1344706 genotype			
	GG	GT	TT	^p^
Gender, male/female	6/5	9/12	9/2	0.12^a^
Handedness, right/left	9/2	18/3	10/1	0.39^a^
Age, years	29.5 ± 10.3	34.2 ± 8.8	31.45 ± 10.1	0.82^b^
Education, years	14.8 ± 1.2	14.5 ± 3	14.4 ± 2.2	0.47^b^
WM, Cowan's K	1.58 ± 0.1	1.54 ± 0.07	1.42 ± 0.1	0.49^c^

*ZNF804A* genotype groups described by gender, handedness, age and education. Statistical significance (p) given for

a χ^2^ test

b ANOVA

c repeated-measures ANOVA.

**Table 2:** Brodmann area (BA), voxel cluster sizes (mm^3^), peak Talairach coordinates for the main effect of emotional face valence

Brain region	BA	Voxels	X	Y	Z	F_(2,80)_	p
Right inferior frontal gyrus	46	174	47	28	12	14.31	<0.00001
Left inferior frontal gyrus	47	138	–28	7	–16	13.80	<0.00001

**Table 3:** Brodmann area (BA), voxel cluster sizes (mm^3^), peak Talairach coordinates for the main effect of WM load

Brain region	BA	Voxels	X	Y	Z	F_(3,120)_	p
Superior temporal gyrus	39	395	53	–57	27	11.49	<0.00001
Right medial frontal gyrus	8	394	2	37	42	11.34	<0.00001
Right lingual gyrus	18	147	2	–80	6	10.63	<0.00001
Right lingual gyrus	18	142	8	–71	–3	9.79	<0.00001
Right inferior parietal gyrus	40	96	60	–32	33	10.39	<0.00001

**Table 4:** Brodmann area (BA), voxel cluster sizes (mm^3^), peak Talairach coordinates for the main effect of rs1344706 genotype

Brain region	BA	Voxels	X	Y	Z	F_(2,40)_	p
Right inferior frontal gyrus	44	45	56	7	21	14.4	0.000019
